# High prevalence of hypertension in an agricultural village in Madagascar

**DOI:** 10.1371/journal.pone.0201616

**Published:** 2018-08-16

**Authors:** Melissa B. Manus, Gerald S. Bloomfield, Ashley Sobel Leonard, Laura N. Guidera, David R. Samson, Charles L. Nunn

**Affiliations:** 1 Duke Global Health Institute, Duke University, Durham, North Carolina, United States of America; 2 Department of Evolutionary Anthropology, Duke University, Durham, North Carolina, United States of America; 3 Department of Medicine and Duke Clinical Research Institute, Duke University, Durham, North Carolina, United States of America; 4 Department of Biology, Duke University, Durham, North Carolina, United States of America; 5 Department of Anthropology, University of Toronto, Mississauga, Canada; Nagoya University, JAPAN

## Abstract

Elevated blood pressure presents a global health threat, with rates of hypertension increasing in low and middle-income countries. Lifestyle changes may be an important driver of these increases in blood pressure. Hypertension is particularly prevalent in African countries, though the majority of studies have focused on mainland Africa. We collected demographic and health data from 513 adults living in a community in rural Madagascar. We used generalized linear mixed models to assess body mass index (BMI), age, sex, and attributes related to household composition and lifestyle as predictors of blood pressure and hypertension. The prevalence of hypertension in this cohort was 49.1% (both sexes combined: N = 513; females: 50.3%, N = 290; males: 47.5%, N = 223). Blood pressure, as well as hypertensive state, was positively associated with age and BMI. Lifestyle and household factors had no significant relationships with blood pressure. The prevalence of hypertension was similar to that found in urban centers of other African countries, yet almost double what has been previously found in Madagascar. Future research should investigate the drivers of hypertension in rural communities worldwide, as well as the lifestyle, cultural, and genetic factors that underlie variation in hypertension across space and time.

## Introduction

Elevated blood pressure affects millions of people around the world [1], with the World Health Organization identifying hypertension as a major global health challenge [2]. Globally, high blood pressure contributes to 92 million disability-adjusted life years (DALYs) and 7.6 million premature deaths [3]. In 2000, hypertension was responsible for 62% of stroke, 49% of ischemic heart disease, and almost 13% of all deaths worldwide [4]. Over ten years later, hypertension remains a leading global risk factor for associated cardiovascular diseases, ranking above tobacco smoking and household air pollution [5].

The prevalence of hypertension in low and middle-income countries (LMICs) is increasing [6, 7]. In fact, approximately two-thirds of the global burden of hypertension is found in developing countries [4]. Compared to other regions of the world, hypertension is most prevalent in Africa [2], where nations have seen marked increases in hypertension over the past half century [8]. For example, a 2012 report documented a 24% increase in the prevalence of hypertension in sub-Saharan Africa from 1998 to 2003 [7]. While it is possible that increased monitoring and improved health data collection could explain the temporal increase in documented hypertension in LMICs, changes in risk factors for hypertension likely underlie some of the observed increase [2].

In LMICs, the prevalence of hypertension tends to be greater in urban areas than rural ones [8, 9]. This difference can be attributed to a myriad of behavioral and lifestyle factors that influence obesity and stress, two known risk factors for hypertension [2, 8–12]. For example, exposure to calorie-rich, processed foods, coupled with a decrease in physical activity, can contribute to weight gain and associated cardiovascular health risks. Similarly, people in urban settings are more likely to experience elevated stress levels related to aspects of industrialization, including increased crime and financial concerns [13]. It is well known that stress is linked to elevated blood pressure [14–16]. A stronger understanding, and ultimately control, of the risk factors for hypertension is important for monitoring a suite of associated public health challenges, as hypertension is a critical contributor to chronic kidney disease [17], cerebrovascular disease, and cardiovascular disease [18].

The relationship between changing lifestyles and health can be understood through the lens of evolutionary medicine, and more specifically, *evolutionary mismatch*—the concept that differences between the current environment and the one in which humans evolved have direct consequences for health [19]. In the context of hypertension in sub-Saharan Africa, a mismatch perspective may help explain why rural communities are susceptible to hypertension in response to elevated stress and changing diets and occupations [20]. Because the pace of lifestyle change in industrialized contexts is much faster than that of biological evolution, populations may become quickly mismatched to novel exposures as countries undergo economic development, the consequences of which are relevant to both global public health and medicine [21]. An evolutionary lens is therefore able to provide richer explanations for disparities in hypertension, such as the increased prevalence in urban populations compared to rural counterparts [13], which is an area that has been identified as requiring further investigation [9].

We investigated body mass index (BMI), age, sex, and blood pressure in an economically developing rural population in Madagascar. This region is increasingly shifting to a reliance on cash crops, such as vanilla, producing a cascade of factors that increase both stress and access to factors of the post-industrial lifestyle, including alcohol and processed foods. More generally, Madagascar is interesting because of its unique ecological and genetic factors, including an admixture of individuals with African and Austronesian ancestry [22]. Thus, this study contributes to others that have focused on lifestyle differences in large urban centers and rural villages on mainland Africa [9, 23], and adds to a smaller number of studies on Malagasy populations [24].

Building on previous research described above, we investigated predictors of blood pressure and hypertension in rural Madagascar. Focusing on key correlates in previous studies, we predicted that elevated blood pressure covaries positively with BMI and age [13, 25], and that males have higher blood pressure than females [25]. We also investigated less commonly studied variables that may be relevant to transitions in this community, including the effects of behavior and stress, predicting that blood pressure covaries positively with tobacco use [13], alcohol use [2, 26], and larger household size (a proxy for stress) [2].

## Materials and methods

This study took place in Mandena, Madagascar (approximately -18°42'00” S 47°50'00” E), an agricultural village of approximately 3,000 people in the SAVA region (an acronym capturing the names of its four major cities: Sambava, Antalaha, Vohémar, and Andapa). Rice production is central to subsistence agriculture in this population, and vanilla is generally sold as a cash crop.

Data were collected over three field seasons in Mandena: (1) Seven weeks from July to August 2015; (2) four weeks from July to August 2016; and (3) four weeks from May to June 2017. Importantly, data collection began at the start of vanilla season, a period when farmers are spending considerable amounts of time monitoring, harvesting, and preparing the crop for sale, including protecting their crops from theft in the field and processing harvested vanilla beans in the village. The SAVA region in particular is tightly connected to global vanilla distribution; as such, communities in the area experience marked seasonal and yearly fluctuations in crop yields and market prices, financial security (i.e. surplus of cash immediately following vanilla sales), and stress.

This study is nested within a larger project that utilized an evolutionary perspective to understanding the relationship between changing lifestyles and health outcomes in this Malagasy village. As new projects were initiated, modifications were made to improve data collection and to collect new health metrics. As such, variation in cohort size and certain data collection procedures exists across the three field seasons, as documented below. All study procedures were approved by Duke University’s Institutional Review Board (Protocol C0848) and by Malagasy health authorities.

The study included a total of 1,142 measurements of blood pressure and other health metrics from 513 adults enrolled over the three field seasons (223 men and 290 women; ages ranged from 18 to 89 years). During each field season, individuals first learned about the project through a town-hall style meeting led by the village president at a central building in Mandena. Interested individuals were encouraged to return to the building in the following days to enroll in the study. To maintain anonymity in the records, each participant was given a unique identification number that was used in all subsequent surveys.

Through a Malagasy translator, each participant provided informed written consent and completed a general health survey, coupled with measurements of height, weight, temperature, blood pressure, and heart rate. All blood pressure and heart rate measurements were taken with the Omron 10 Series Upper Arm Blood Pressure Monitor (Omron Healthcare, Inc.). Individuals were required to sit for at least five minutes before the first blood pressure reading, and at least five minutes separated a total of up to three different readings. We used a standard bathroom scale that was purchased in Madagascar to measure weight, and a tape measurer adhered to a vertical wooden beam in the central building to measure height. Height and weight measurements were used to calculate BMI as weight in kilograms divided by the square of height in meters. Following standard guidelines, individuals were categorized as: underweight <18.5 kg/m^2^; normal ≥18.5 to <25 kg/m^2^; overweight 25–30 kg/m^2^; obese ≥30 kg/m^2^ [27]. Any participants with clinically elevated measurements were instructed to speak with a Malagasy nurse who worked alongside our team in Mandena.

Comparison of data collection across the three study years are shown in Table 1. In 2015, blood pressure was recorded once per participant; in 2016 and 2017, up to three readings were taken per participant. We recorded multiple measurements to assess changes in blood pressure over repeated readings, with declines indicating a decrease in blood pressure over longer sedentary time and/or reduced stress associated with the procedure (S1 Fig). In 2016, 23% of 142 participants had their blood pressure recorded in their homes, while all other measurements were collected at the central building, including all blood pressure readings in 2015 and 2017.

**Table 1:** Data collection from 2015–2017.

Category	2015	2016	2017
Blood pressure readings per participant	1	Up to 3	Up to 3
Blood pressure measurement location	Central building	Central building and participants’ home	Central building
Weeks of data collection	7	4	4
BMI data available	Yes	Yes	Yes
Alcohol and tobacco use data available	Yes (N = 47)	No	No
Household data available	Yes (N = 47)	No	No

We used household size (number of people) as a proxy for stress, as assessed using the general health survey in 2015. We also collected data on tobacco and alcohol usage in the previous week (yes/no).

We used generalized linear mixed models (GLMM) to analyze blood pressure, with one reading per participant from 2015 and all readings from participants in 2016 and 2017 (N = 1,142). To incorporate the decrease in blood pressure over repeated readings, we included individual ID and measurement (first, second, or third) as random effects, and BMI, age, and sex as fixed effects. We then used model selection and averaging to predict both systolic (SBP) and diastolic (DBP) blood pressure as continuous variables. All analyses were run in R [28].

We were also interested in investigating the relationship between hypertension and factors related to transitions toward industrialization. Using the ACC/AHA guidelines outlined in Whelton et al. [29], we defined hypertension as systolic blood pressure ≥ 130 mm Hg or diastolic blood pressure ≥ 80 mm Hg. It is worth noting that due to their recent publication, these guidelines are not used in many previous studies (hypertension was previously defined as systolic blood pressure ≥ 140 mm HG or diastolic blood pressure ≥ 90 mm Hg). Blood pressure categories were used to describe cohort demographics and to generate a binary classification for each individual (i.e., hypertension present or absent), which was then used in subsequent analyses. This was made difficult by having only one reading per individual in 2015, coupled with the aforementioned evidence that blood pressure declined with reading number. Thus, to create a better estimate of blood pressure in 2015, we constructed a linear model that used the first blood pressure reading to predict the third (SBP: intercept = 10.918, β = -0.876, t-value = -25.1, p<0.001, df = 130; DBP: intercept = 8.268, β = -0.874, t-value = -14.4, p<0.001, df = 130). We then used the “predict” function to predict the third blood pressure reading for each individual in the 2015 cohort. The complete set of third blood pressure readings (i.e. the predicted readings for 2015 and the measured readings for 2016 and 2017; N = 513) was then used to determine hypertensive state (presence or absence), with non-hypertensive individuals assigned to “normal” or “elevated” state. Because the predict method makes a conservative assumption regarding the third reading, the presence of hypertension based on predicted third readings is unlikely to be overestimated. With this method, only eight out of 203 (3.9%) individuals were re-classified from hypertensive (first reading) to non-hypertensive (predicted third reading). We re-ran models without the predicted data and obtained similar results, further supporting the validation of this statistical approach. A GLMM was then constructed to predict hypertensive state with a binomial link (i.e. a logistic regression), using individual ID as a random effect, and BMI, age, and sex as fixed effects.

Finally, we investigated lifestyle and household predictors based on data from 2015 (N = 47). We used model selection and averaging of generalized linear models to predict SBP and DBP based on BMI, age, sex, alcohol consumption and tobacco usage in the previous week (y/n), and household size (number of people).

## Results

**Fig 1:**
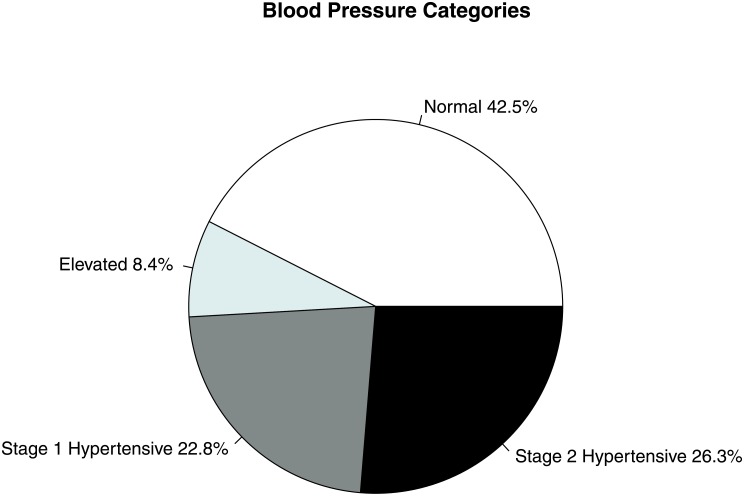
Distribution of the study population into blood pressure categories (both sexes combined). We found that nearly one-half (49.1%) of the population is hypertensive, based on criteria from Whelton et al. [29].

**Fig 2:**
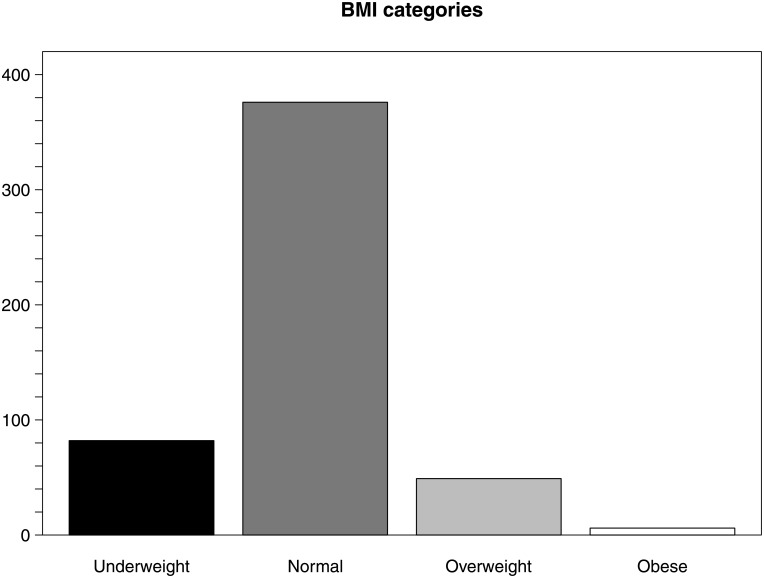
Histogram of individuals in each BMI category. Underweight (<18.5 kg/m^2^) = 16%; normal (≥18.5 to <25 kg/m^2^) = 73.3%; overweight (25–30 kg/m^2^) = 9.5%; obese (≥30 kg/m^2^) = 1.2%.

**Fig 3:**
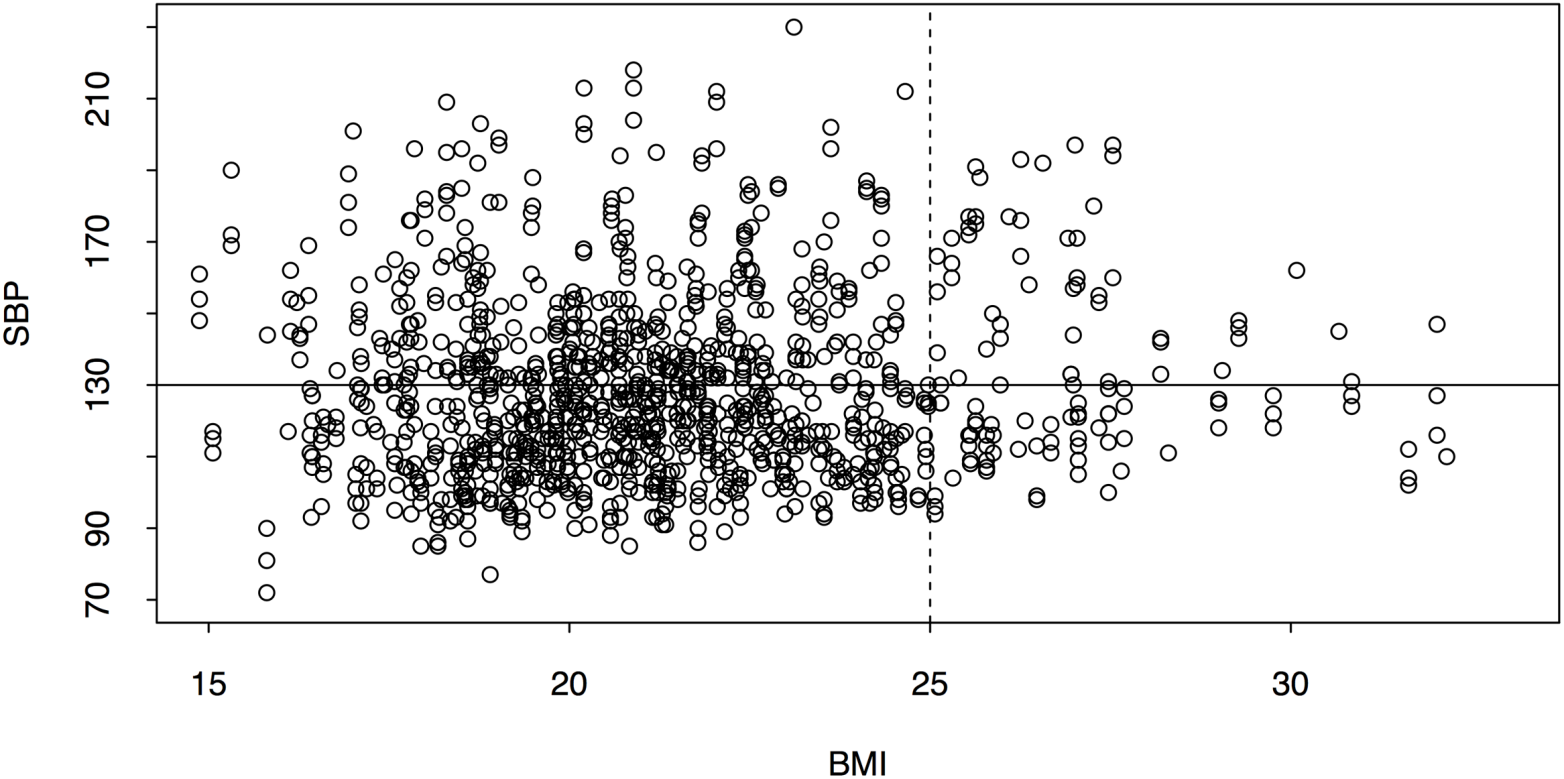
Blood pressure and BMI. The solid lines separate hypertensive and non-hypertension individuals, while the dashed lines demarcate overweight and obese individuals from those who are normal or underweight. The top left quadrant includes individuals who are under/normal weight and hypertensive, and the bottom right quadrant includes those who are overweight/obese and non-hypertensive.

Of the 513 participants, we identified 49.1% as hypertensive (Stages 1 and 2 combined). Of those that were not hypertensive, we found that 8.4% had elevated blood pressures, with only 42.5% having clinically normal measurements (Table 2 and Fig 1). Overall, 73.3% of individuals presented with normal BMIs (Figs 2 and 3), while 16% of individuals were underweight and only 10.7% were overweight or obese. Overall cohort demographic data can be found in the Supporting Information.

**Table 2:** Cohort demographics across different blood pressure categories [29].

Category	SBP	DBP	Both sexes combined (N = 513)	Men (N = 223)	Women (N = 290)
Normal*	<120 mm Hg	<80 mm Hg	218 (42.5%)	91 (40.8%)	127 (43.8%)
Elevated*	120–129 mm Hg	<80 mm Hg	43 (8.4%)	26 (11.7%)	17 (5.9%)
Stage 1 Hypertension**	130–139 mm Hg	80–89 mm Hg	117 (22.8%)	52 (23.3%)	65 (22.4%)
Stage 2 Hypertension**	≥140 mm Hg	≥90 mm Hg	135 (26.3%)	54 (24.2%)	81 (27.9%)

Values indicate prevalence and percentage of column total (in parentheses).

* Both systolic and diastolic measures must fall within the listed range.

** Either systolic or diastolic measurement need fall within the listed range.

In statistical analyses of SBP and DBP, we found that both were positively predicted by age and BMI, but not by sex (Table 3 and S2 Fig). Similarly, hypertensive state was positively predicted by age and BMI, while effects of sex were non-significant (Table 3). A greater proportion of women (50.3%) were hypertensive compared to men (47.5%) (Table 2).

**Table 3:** Predictors of blood pressure across all study years, controlling for individual and reading number.

*Systolic blood pressure (continuous)*				
**Variable**	**Estimate**	**z-value**	**Lower CI**	**Upper CI**
BMI	0.178	4.623	0.103	0.254
Age	0.429	11.070	0.353	0.505
Male	0.075	1.937	-0.001	0.150
*Diastolic blood pressure (continuous)*				
**Variable**	**Estimate**	**z-value**	**Lower CI**	**Upper CI**
BMI	0.082	2.132	0.007	0.157
Age	0.280	7.189	0.204	0.356
Male	-0.034	0.863	-0.111	0.043
*Hypertensive state (categorical)*				
**Variable**	**Estimate**	**z-value**	**Lower CI**	**Upper CI**
BMI	0.451	2.160	0.041	0.862
Age	1.615	6.468	1.126	2.105
Male	-0.091	0.435	-0.499	0.318

Further analyses revealed no significant effect of lifestyle and stress-related factors that we investigated (i.e., household size and tobacco and alcohol usage), although sample sizes were smaller than the overall cohort. Full results and demographic data are provided in the Supporting Information.

## Discussion

The overall prevalence of hypertension in this rural Malagasy community was nearly 50%. This is higher than what is reported in many rural communities on mainland Africa [12, 13, 26, 30], and interestingly, similar to rates observed in urban settings in other African countries [9, 31]. It should be noted that our categorization of hypertension follows the ACC/AHA guidelines recently published in Whelton et al. [29, 32], while much of the existing literature, including another study in Madagascar [24, 32], used the previous standards for the diagnosis of hypertension (i.e., SBP≥140 mmHg or DBP≥90 mmHg). Based on previous guidelines [32], we would have reported the prevalence of hypertension in Mandena to be 26%. This rate of hypertension is similar to findings from a number of studies that used the previous guidelines—including in rural Madagascar (27%) [24], Ghana (24.1% and 27% [13, 33]), Nigeria (19.3%), and Kenya (21.4%) [34]. Thus, use of the new ACC/AHA guidelines in other LMICs is likely to reveal the high rates of hypertension found in our study. As the new guidelines reflect health concerns that can occur at lower blood pressures, re-evaluating hypertension in these communities can ensure more robust surveillance of associated morbidities, such as cardiovascular and chronic kidney diseases [17, 18].

We found that BMI covaried positively with measures of SBP and DBP. These patterns mirror previous studies of blood pressure in rural communities [13, 33, 35, 36]. It is likely that cultural and behavioral changes in Mandena contribute to this observed trend. For example, increased access to processed foods and soft drinks can lead to greater BMI and higher blood pressure, an association often used to explain the growing rates of hypertension in urban centers of developing countries where these dietary items are common [7]. Anecdotal observations by the authors who performed data collection suggest an increase in processed foods and soft drinks in Mandena, as well as transportation infrastructure that could promote more sedentary lifestyles (i.e. cars, motorcycles, and buses). While BMI measurements in this population are relatively low (89% of individuals were classified as underweight or normal weight), similar associations between BMI and blood pressure have been reported in other lean populations [37, 38]. An evolutionary perspective can help to rigorously evaluate factors that may be directly affected by transitions toward industrialization (i.e., where the opportunity for mismatch is high), including diet, physical activity level, and stress.

While there is a positive association between BMI and hypertension, it is interesting that only 1.2% of individuals are classified as obese. It is possible that additional metrics related to body mass and metabolism, such as waist circumference, would better elucidate patterns in this population [39]. Because the majority of hypertensive individuals in our study are not overweight or obese (Fig 3), future research should explore the disconnect between expected (overweight) and observed (lean) phenotype in those presenting with hypertension.

Older age significantly predicted hypertensive state, a finding that is in line with many other studies [9, 12, 25, 30, 36]. This is relevant to global public health initiatives more generally, as programs that successfully alleviate mortality (i.e., via improved nutrition, vaccination, or access to healthcare) may in turn be challenged by an increasing incidence of hypertension due to the greater number of individuals that reach older age [24]. Our data indicate clinically elevated blood pressures in the younger demographic, indicating that it is not solely the elderly who are at risk for elevated blood pressure (S2 Fig) [24, 40]. Understanding behavior and health status among the younger demographic may help elucidate drivers of the progression to hypertension.

Contrary to our predictions, sex did not have a significant effect on hypertension, even within specific age groups (data not shown). While previous studies have found higher blood pressure in men than women [25], studies in rural Madagascar and Ghana also failed to document a significant association between sex and hypertension [24, 33, 41]. It may be that cultural and livelihood practices contribute to the lack of significant sex differences. For example, both men and women work in the agricultural fields in Mandena, which may yield similar levels of cardiovascular health in both sexes.

To our knowledge, this is only the second study to examine blood pressure in rural Madagascar. A previous study also found a positive relationship between age and hypertension, and raised concerns over the risk of a hypertension epidemic in Madagascar’s progressively aging society [24]. Strengths of our study include its relatively large sample size, with data collection spanning three years, and the inclusion of additional lifestyle measures. Possible limitations include biased sampling, as unemployed and/or unhealthy individuals may be more likely to visit our health clinic during the day compared to their employed, healthy counterparts. Similarly, all data were collected during the austral winter, which may reflect a seasonal bias in activity patterns or stress levels. We also note that stages of hypertension were determined using Western standards, which may not capture important biological factors of this genetically unique, non-Western population [25, 42]. Future work in this population should consider genetic factors that can influence high blood pressure, particularly as they relate to individuals of African and Austronesian ancestry (i.e. the ancestral populations of the modern day Malagasy) [43]. It would also be interesting to assess the influence of comorbidities, such as communicable diseases, on blood pressure—including the intriguing potential tradeoff between elevated blood pressure and protection against malaria [44].

In conclusion, we discovered a remarkably high prevalence of hypertension in an agricultural community in rural Madagascar. Our research raises a number of questions to address in future studies, including research aimed at understanding the drivers of hypertension throughout the life course. Future studies in this region could incorporate data on dietary sodium intake and family history of hypertension, along with metrics of stress, including stress associated with vanilla production. More generally, our study contributes to the growing body of literature that calls for making the study of hypertension in rural communities a priority in global health research [8, 10, 30, 35, 45].

## Supporting information

S1 Fig Comparison of the first and third blood pressure readings from 2016 and 2017. The third measurement is lower than the first.
(EPS)

S2 Fig SBP (R^2^ = 0.186, p<0.001) and DBP (R^2^ = 0.067, p<0.001) increases with age. The dashed line indicates the minimum blood pressure value for hypertension.
(PDF)

S1 Table Cohort demographics, where SBP and DBP are the third readings. (PDF)

S2 Table Predictors of blood pressure, as related to lifestyle and household attributes, from 2015 data (N = 47). (PDF)

S3 Table Demographic data, as related to lifestyle and household attributes. (PDF)

S1 Dataset (XLSX)
