# Supplementary figures and images for: High prevalence of hypertension in an agricultural village in Madagascar
Source: PLoS One. 2018 Aug 16;13(8):e0201616. doi: 10.1371/journal.pone.0201616 (PMC6095505; doi:10.1371/journal.pone.0201616)

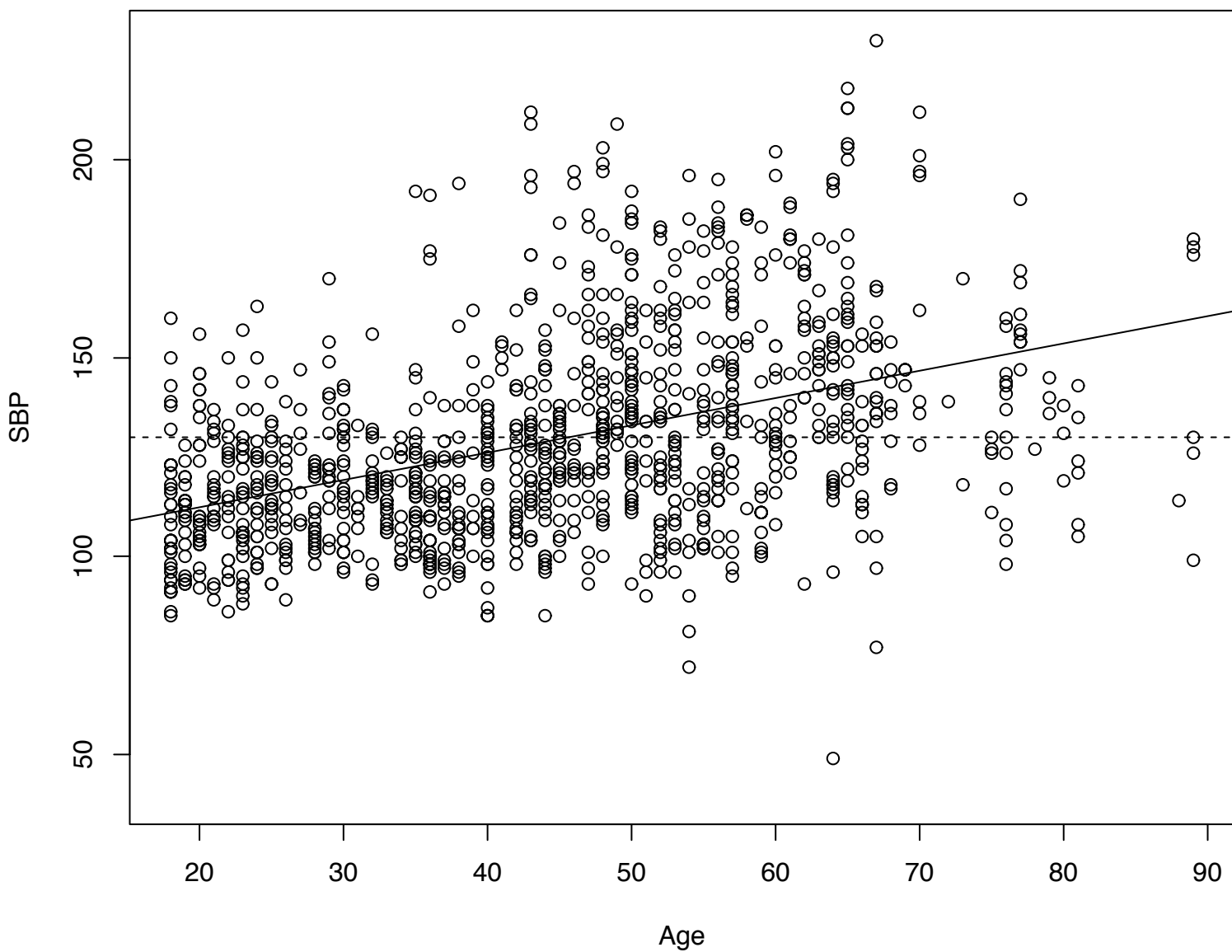

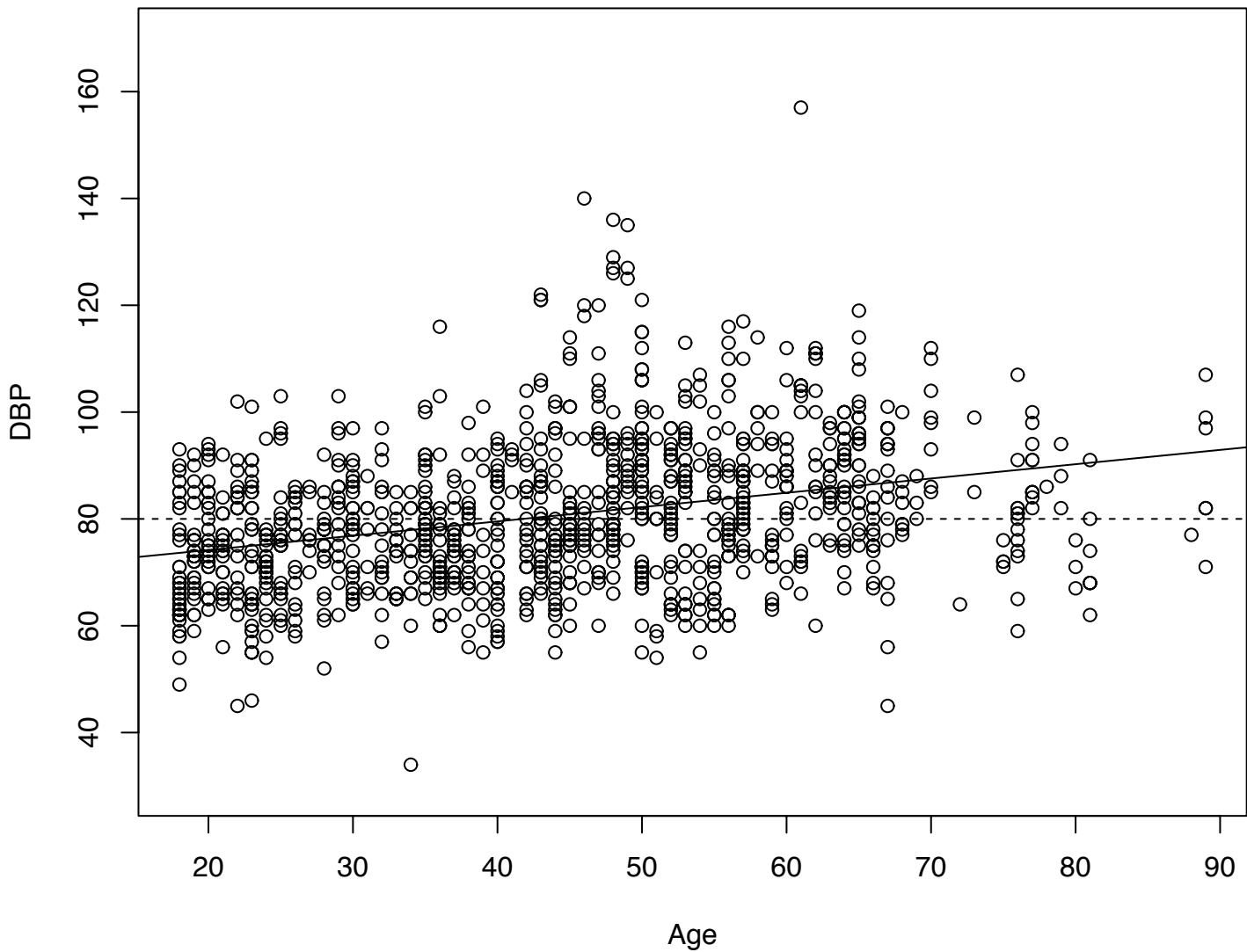

Supplement: S2 Fig — The dashed line indicates the minimum blood pressure value for hypertension. (PDF) [file pone.0201616.s002.pdf]
