# Supplementary material for: High prevalence of hypertension in an agricultural village in Madagascar
Source: PLoS One. 2018 Aug 16;13(8):e0201616. doi: 10.1371/journal.pone.0201616 (PMC6095505; doi:10.1371/journal.pone.0201616)
Supplement: S1 Table — (PDF) [file pone.0201616.s003.pdf]

Both Sexes Combined (N=513)					
**Variable**	**Mean**	**Median**	**Std Dev**	**Minimum**	**Maximum**
Age (years)	43.62	43	16.11	18	89
Height (cm)	158	157	8.14	131	198
Weight (kg)	53.16	53	8.07	34	85.20
BMI	21.31	20.96	2.97	13.91	32.16
SBP	124.60	120	23.71	72	212
DBP	79.21	77.5	13.85	38	157
Men (N=223)					
**Variable**	**Mean**	**Median**	**Std Dev**	**Minimum**	**Maximum**
Age (years)	44.20	45	15.84	18	81
Height (cm)	163.8	164	7.12	145	198
Weight (kg)	55.06	55	7.04	37	74.50
BMI	20.52	20.69	2.17	13.91	27.04
SBP	125.70	122	20.94	72	203
DBP	78.50	77	13.04	45	157
Women (N=290)					
Variable	**Mean**	**Median**	**Std Dev**	**Minimum**	**Maximum**
Age (years)	43.18	42	16.33	18	89
Height (cm)	153.60	154	5.82	131	172
Weight (kg)	51.74	51	8.51	34	85.20
BMI	21.91	21.64	3.32	14.87	32.16
SBP	123.90	118	25.60	85	212
DBP	79.75	78	14.43	38	156
