# Supplementary material for: High prevalence of hypertension in an agricultural village in Madagascar
Source: PLoS One. 2018 Aug 16;13(8):e0201616. doi: 10.1371/journal.pone.0201616 (PMC6095505; doi:10.1371/journal.pone.0201616)
Supplement: S2 Table — (PDF) [file pone.0201616.s004.pdf]

Systolic blood pressure (continuous)

Variable	Estimate	z-value	Lower CI	Upper CI
BMI	0.137	0.835	-0.185	0.460
Age (years)	0.256	1.565	-0.065	0.576
Male	0.129	0.769	-0.200	0.458
Household size	0.278	1.738	-0.036	0.592
Alcohol in last week (y/n)	-0.063	0.383	-0.382	0.257
Tobacco in last week (y/n)	-0.090	0.577	-0.394	0.215

Diastolic blood pressure (continuous)

Variable	Estimate	z-value	Lower CI	Upper CI
BMI	0.084	0.494	-0.250	0.418
Age (years)	0.008	0.046	-0.323	0.339
Male	-0.029	0.177	-0.357	0.298
Household size	0.148	0.880	-0.182	0.477
Alcohol in last week (y/n)	0.124	0.746	-0.201	0.449
Tobacco in last week (y/n)	-0.206	1.294	-0.517	0.106
