# Supplementary material for: High prevalence of hypertension in an agricultural village in Madagascar
Source: PLoS One. 2018 Aug 16;13(8):e0201616. doi: 10.1371/journal.pone.0201616 (PMC6095505; doi:10.1371/journal.pone.0201616)
Supplement: S3 Table — (PDF) [file pone.0201616.s005.pdf]

Both Sexes Combined (N=47)					
**Variable**	**Mean**	**Median**	**Std Dev**	**Minimum**	**Maximum**
Age (years)	45.62	47	15.91	20	76
Height (cm)	156.8	156	10.59	109	177.50
Weight (kg)	51.54	51	7.79	38	73
BMI	21.24	20.69	5.31	16.65	53.03
SBP	125.50	121	21.56	93	182
DBP	79.36	80	8.85	60	100
Household size	4.57	4	2.58	1	10
Men (N=20)					
**Variable**	**Mean**	**Median**	**Std Dev**	**Minimum**	**Maximum**
Age (years)	42.65	43	16.69	20	75
Height (cm)	161.30	164	13.93	109	177.50
Weight (kg)	56.45	57	6.84	6	73
BMI	22.49	21.03	7.58	16.65	53.03
SBP	127.10	121.50	18.14	95	181
DBP	79.05	79	7.62	66	94
Household size	3.75	3	2.34	1	10
Women (N=27)					
**Variable**	**Mean**	**Median**	**Std Dev**	**Minimum**	**Maximum**
Age (years)	47.81	49	15.25	20	76
Height (cm)	153.50	152.50	5.45	146	169
Weight (kg)	47.91	47	6.39	38	63
BMI	20.32	20.44	2.42	17.54	26.91
SBP	124.40	120	24.05	93	182
DBP	79.59	80	9.79	60	100
Household size	5.22	5	2.62	1	10
